# Twenty years since the 2004 European Union enlargement: what does it mean for health?

**DOI:** 10.1093/eurpub/ckae071

**Published:** 2024-04-15

**Authors:** Rok Hrzic, Helmut Brand

**Affiliations:** Department of International Health, Care and Public Health Research Institute—CAPHRI, Maastricht University, Maastricht, The Netherlands; Department of International Health, Care and Public Health Research Institute—CAPHRI, Maastricht University, Maastricht, The Netherlands; Department of Health Policy, Prasanna School of Public Health, Manipal Academy of Higher Education, Manipal, India

This year marks two decades since the ‘big bang’ enlargement of the European Union (EU) in 2004. The monumental event reshaped the geopolitics of Europe, with 10 new countries joining the bloc, 7 of which lay behind the former Iron Curtain. (Slovenia was a former republic of the non-aligned Yugoslavia). The accession process was a mix of optimism due to the perceived victory of Western values such as liberal democracy and free markets and apprehension due to the vast differences between the old and new EU members, including differences in health. At the time of accession, the new member states lagged the EU-15 in life expectancy at birth.^1^ Twenty years later, this gap persists and has ballooned—hopefully temporarily—in the aftermath of the COVID-19 pandemic. While most EU-15 countries experienced a dip in life expectancy in 2020 but recovered in 2021, most new Eastern European member states saw a further deterioration in 2021. Slovakia, for example, saw an almost three-year decline.^2^ The reasons for this require further investigation but generally indicate a continuing discrepancy in health system performance and resilience.

The persistent differences in population health and health system performance are surprising, given the efforts expended during and after the accession process. The EU provided technical assistance and invested tens of billions in the accession countries. However, a closer inspection indicates that the efforts were too modest and narrow to reshape population health in all accession countries. Comparing the 2004 EU enlargement to the German reunification, where a colossal multi-trillion Euro investment contributed to largely eliminating the East–West mortality gap, the 2004 EU accession portfolio of investments was about 100-fold smaller per citizen. In addition, the limited mandate of the EU in health and social policy, two critical determinants of population health, means that the transfer of critical institutions that could lead to rapid improvements in health was limited.^3^ These discrepancies in the scale of investment and institutional transfer are understandable, given the differences in political context and available instruments. Still, they play an essential role in understanding the observed enduring differences in health across the EU.

It is crucial to remain vigilant of the health disparities across the EU, but focusing on the differences between old and new member states may be misleading. This approach overlooks the significant variations among the new member states. Consider Estonia, for example. Since 2000, Estonia has experienced the fastest life expectancy growth in the EU, driven by remarkable reductions in cardiovascular deaths. Effective and timely healthcare system reforms strengthening primary care and a rigorous alcohol policy are the most likely drivers of this achievement. The EU supported this success through investments that improved critical healthcare infrastructure, bridged a public health funding shortfall during the Great Recession and supported actions against harmful alcohol use.^4^ This indicates that the tools at the EU’s disposal *can* help transform population health, but only if they are carefully aligned with member state’s health challenges, vision and capabilities. In other words, EU accession is insufficient to improve population health outcomes. Instead, it is critical to develop national health policies that respond to the unique national context *and* that can synergize with what the EU offers—which is by no means a simple feat. To support all member states in this endeavour, we need research that helps us understand better how EU policies interact with different national and regional contexts and consider whether new, more flexible policy tools are required.

The future of European public health depends on even closer collaboration between EU institutions and the member states. Disagreements on tackling current and future challenges, ranging from geopolitics and war to migration, climate change and LGBTQIA+ rights, show clearly that this collaboration cannot be taken for granted. EU member states, new and old, seek to manoeuvre their way to continued relevance and influence, sometimes at the expense of European unity. It is heartening, however, to see steps in the direction of re-doubled collaboration in health. Consider the emergence of the European Health Union, where EU institutions and member states alike have recognized that future health security depends on solidarity and cooperation in procurement and coordinated preparedness and response measures, or consider the recent breakthrough in the negotiations on the European Health Data Space, where the emerging consensus promises to support citizens, patients, researchers and entrepreneurs in leveraging the wealth of health data across the EU. While there may be much we disagree on, there is a clear consensus on the continued importance of good health across the EU. As the turbulent 21st century unfolds, European public health^5^—the intersection of EU action and public health—promises to be a dynamic and critical focus for public health researchers and policymakers.

*Conflicts of interest*: None declared.
